# Challenges for remote patient monitoring programs in rural and regional areas: a qualitative study

**DOI:** 10.1186/s12913-025-12427-z

**Published:** 2025-03-13

**Authors:** Joel Fossouo Tagne, Kara Burns, Teresa O’Brein, Wendy Chapman, Portia Cornell, Kit Huckvale, Ishaan Ameen, Jaclyn Bishop, Alison Buccheri, Jodie Reid, Anna Wong Shee, Marc Budge, Catherine E. Huggins, Anna Peeters, Olivia Metcalf

**Affiliations:** 1 Centre for Digital Transformation of Health, University of Melbourne, Level 8 – Melbourne Connect, 700 Swanston St., Melbourne, VIC 3010 Australia; 2 Western District Health Service, Hamilton, Australia; 3 East Grampians Health Service, Ararat, Australia; 4 Colac Area Health, Colac, Australia; 5 Barwon Health, Geelong, Australia; 6 Grampians Health, Ballarat, Australia; 7 Bendigo Health, Bendigo, Australia; 8 Institute for Health Transformation, Deakin University, Geelong, Australia; 9 Deakin Rural Health, Warrnambool, Australia; 10 Monash Rural Health Bendigo, Monash University, Bendigo, Australia

**Keywords:** Remote patient monitoring, Rural healthcare, Digital health equity

## Abstract

**Background:**

Access to healthcare significantly influences health outcomes, and rural, regional and remote populations face greater challenges in accessing healthcare than urban populations. Digital health tools, such as remote patient monitoring (RPM), have significant potential to address these healthcare challenges, yet there is little research on the facilitators and barriers of RPM in these regions.

**Aim:**

This study aims to identify and understand the facilitators and barriers healthcare staff face implementing RPM in rural and regional Australia, with focus on challenges that arose after the onset of the COVID-19 pandemic.

**Methods:**

Semi-structured focus groups were conducted with healthcare professionals from publicly funded health services in western rural and regional Victoria, Australia. An open-ended interview guide based on the Consolidated Framework for Implementation Research (CFIR) was used to identify key themes and strategies for effective RPM implementation. The analysis considered barriers and facilitators at micro, meso, and macro levels.

**Results:**

Several barriers to RPM implementation were identified across different levels: (1) Micro-Level Factors, such as perceived low digital literacy and language barriers among individuals; (2) Meso-Level Factors, including disparities in IT infrastructure and device availability, limited training opportunities, and the need for enhanced governance within healthcare settings; and (3) Macro-Level Factors, encompassing evolving funding models and the reliability of service providers. Despite these challenges, participants acknowledged potential benefits such as improved technological interoperability, enhanced community engagement, and a data-driven approach to quality improvement. Importantly, a flexible, tailored RPM approach to accommodate specific rural and regional needs was deemed valuable.

**Conclusion:**

Effective RPM deployment in rural and regional areas is viewed by health professionals as crucial for bridging healthcare divides. However, if strategies developed for urban settings are not recalibrated to address rural challenges, the risk of RPM failure may escalate. Future initiatives must prioritize region-specific strategies and policy reforms aimed at ensuring equitable digital infrastructure and financial resource allocation to enhance healthcare access in rural and regional settings. This approach may ensure that RPM solutions are both adaptable and effective, tailored to the unique needs of each community.

**Supplementary Information:**

The online version contains supplementary material available at 10.1186/s12913-025-12427-z.

## Background

Access to medical care is a critical determinant of health [1]. People living in rural and regional areas experience a higher burden of disease, and face challenges to accessing healthcare such as limited services and ongoing healthcare professional shortages [2, 3]. Research shows that the further people live from healthcare facilities the poorer their health outcomes [4]. In Australia, people living in rural and regional areas have been recognised as a priority population for addressing health inequity [2, 5].

Digital health tools could help address this disparity by enabling access to health services in rural and regional areas [6]. For example, since the onset of the COVID-19 pandemic, digital health innovations were rapidly implemented and scaled to provide solutions to the challenges of delivering health care when in-person visits were restricted for infection control [7, 8]. The rapid expansion of digital health provided people with ongoing access to vital health services while minimizing their potential exposure to infection. However, despite the potential for digital health in rural and regional areas, emerging research indicates lower uptake among these populations compared to their metropolitan counterparts [6, 9, 10]. A 2023 study investigating consumer preferences for telehealth reported that consumers living in rural and regional Australia preferred travelling to see their doctor and were less likely to engage with any telehealth modalities [11]. Another Australian study noted “one size does not fit all” and that consumers value the availability of telehealth and having the choice and flexibility to use telehealth when appropriate [12].

Remote patient monitoring (RPM) is a form of telehealth delivery [3]. While telehealth is primarily designed to provide clinical consultation and overcome geographical barriers, the primary goal of RPM allows the continuous monitoring of health conditions for people living in the community using digital technology to collect health data from a patient from a location outside the healthcare system [13]. While RPM technology has the potential to enhance patient satisfaction, reduce hospital readmissions, and improve the management of chronic diseases such as in heart disease [14], disparities in RPM access could leave some populations behind, leading to poorer care coordination, unnecessary hospital expenditures, and significant health inequalities [1, 3, 14]. Research has shown that RPM is less likely to be implemented in rural and regional healthcare settings, particularly in lower socio-economic areas, due to factors such as inadequate infrastructure, limited funding, and lower levels of digital literacy among patients [3]. A 2024 review examined the barriers and facilitators of digital health technology implementation, including remote monitoring, remote consultations, and digital care platforms, from the perspective of healthcare professionals [15]. However, this review did not specifically address the unique challenges and contextual factors influencing RPM in rural and regional settings [15]. Additionally, the studies included in the review were conducted between 2012 and 2021, highlighting the need for more recent data to reflect the evolving landscape of digital health technologies.

Since the onset of the COVID-19 pandemic, studies have explored patient and healthcare staff engagement and satisfaction with RPM programs, both within Australia [16, 17] and globally [18–21]. Many of these studies focused specifically on RPM programs designed for monitoring patients diagnosed with COVID-19, often within metropolitan settings. There is a scarcity of research on the utilization of RPM in rural and regional areas. Our study addresses this gap by examining RPM implementation in these areas, encompassing various health conditions, including but not limited to those impacted by the COVID-19 pandemic [22]. Jang-Jaccard et al. [23] and Newman et al. [24] explored telehealth barriers in rural Australia, highlighting the challenges in digital health implementation. Building upon this foundation, this study extends this conversation to the realm of RPM, by examining the perspectives of healthcare staff working in Western Victoria, which may offer insights applicable to other regions in Australia, regarding the use, drivers, and limitations of RPM and its impact on health service delivery. Guided by the Consolidated Framework for Implementation Research (CFIR) as a conceptual lens, this framework may be instrumental in identifying and addressing the intricate barriers and facilitators influencing the adoption of RPM in rural and regional healthcare settings [25]. By capturing the evolving landscape of digital health since the onset of the COVID-19 pandemic, this study provides timely insights into RPM usage. Moreover, examining RPM use across a variety of health conditions may enhance the scope of understanding [26]. Recent reviews underscore the importance of healthcare professional perspectives in digital health implementation [15]. Additionally, this study addresses the need for actionable recommendations to promote digital health equity and improve access to RPM programs for vulnerable populations, such as older adults, in these regions [1, 3, 27].

Given these objectives, the primary research question guiding this study is: What are the barriers and facilitators faced by healthcare providers in rural and regional Australia in implementing Remote Patient Monitoring (RPM), particularly in the context of the challenges that arose after the onset of the COVID-19 pandemic?

## Methods

### Study design

The research design was a qualitative study based on semi-structured focus groups with staff from five health services in regional Victoria (Australia).

### Recruitment

Ethical approval for this study was granted by the Barwon Health Human Research Ethics Committee (BHHREC; 21/12) and site-specific approval was granted at all participating sites, aligning with the National Statement on Ethical Conduct in Human Research (2023) to ensure participant protection and respect throughout the research.

Recruitment strategies varied across the five participating health services, which were selected as part of the DELIVER research program. This program, funded by the Medical Research Future Fund (MRFF) through the Rapid Applied Research Translation Grant (RARUR000072), is a collaborative effort involving rural and regional health services in Western Victoria, led by Western Alliance, Deakin University, and other academic partners, including the Digital Health Validitron at the University of Melbourne. These health services assisted in distributing invitation letters to their staff. A mix of personal outreach, colleague nominations, and snowball sampling was used to identify potential participants. Written informed consent was obtained from all participants. Prior to the semi-structured focus groups, potential participants received a plain language statement (PLS) explaining the study’s purpose, procedures, and voluntary nature. They were given time to consider the PLS before signing a consent form, which was collected by the researchers. Participants committed 90 min to semi-structured focus groups discussing existing RPM programs and their development, without soliciting personal or sensitive information. Participation was unlikely to cause adverse events; however, the Participant Information and Consent Form (PICF) outlined support procedures for any distress. Participants could withdraw at any time, and their contributions up to the point of withdrawal were included in the aggregated research results. To withdraw, participants notified a research team member and signed the withdrawal of consent form.

### Setting and participants

One semi-structured focus group was conducted for each participating health service. Participants were carefully selected based on their knowledge of RPM programs and resources and included a mix of clinical and non-clinical staff, representing a spectrum of roles from novice professionals to senior management, ensuring a comprehensive range of perspectives on RPM implementation. Led by researchers, these focus groups were conducted across the five participating health services categorized according to the modified Monash (MM) category, ranging from MM2 to MM5 [28]. The MM model measures rural remoteness and population size on a scale from MM1 (major city) to MM7 (very remote). These organizations vary in size, ranging from small rural hospitals to larger regional health services. Collectively, they provide a broad range of healthcare services, including inpatient care, outpatient services, primary care, and allied health. A total of 48 participants took part in the study, with 29 identifying as having a clinical role (e.g., nurses, allied health professionals, and physicians) and 19 as having a nonclinical role (e.g., managers, IT specialists, and administrative staff). The number of participants in each focus group was as follows: Focus Group 1–5 had 6, 14, 10, 10, and 8 participants, respectively. The semi-structured focus groups were held both remotely and in-person, based on participants’ preferences and availabilities. This approach enabled the collection of diverse insights into RPM implementation, reflecting a broad range of professional expertise and organisational contexts.

### Data collection

Semi-structured focus groups were conducted using open-ended questions to explore RPM program priorities, feasibility, barriers, and the broader digital maturity of health services. The semi-structured focus groups were facilitated by KB, KH and OM, researchers with expertise in qualitative methods and digital health. The research team included both clinical and non-clinical members to ensure diverse perspectives and minimize potential biases. Discussions were recorded using both audiovisual methods and transcripts for subsequent analysis.

The interview guide was specifically developed for this study and structured to examine barriers and facilitators across CFIR’s five domains. This guide has not been previously published. An English-language version, including a detailed mapping of questions to CFIR domains, is provided in Supplementary file 1.

### Data analysis

We applied a deductive approach followed by an inductive analysis to analyse the qualitative data using NVivo 12 (Lumivero) software. This combined approach enabled us to align the data with an existing framework (CFIR) and then synthesize the findings to develop a nuanced understanding of the phenomenon.

The CFIR framework identifies five major domains that influence implementation outcomes: Intervention Characteristics, Outer Setting, Inner Setting, Characteristics of Individuals, and Process [25]. These domains provide a comprehensive approach to assessing barriers and facilitators at multiple levels.

Two members of the project team (JF, TOB) independently assigned codes and subcodes to the data, including semi-structured focus group transcripts and field notes, grouping them into themes relevant to the research questions. From the outset, field notes and transcripts were analysed together to ensure a comprehensive understanding of the data. Following the initial coding phase, the researchers met to discuss and reach consensus on the identified codes and emergent themes, resolving any discrepancies through consultation with a third researcher (OM). Subsequently, an in-depth thematic analysis was performed, producing detailed summaries that encapsulated the perceptions and experiences shared by the provider informants.

To validate and contextualize the findings, interim results from each semi-structured focus group were iteratively presented to the respective participating provider groups as part of a “member checking” process. This iterative process ensured that the findings accurately represented participant perspectives and were grounded in the context of the studied settings.

Finally, we generated a comprehensive summary of the data, reflecting on its practical implications. The semi-structured focus groups were concluded when no new insights emerged, indicating data saturation and a thorough exploration of the research questions.

## Results

### Overview of RPM programs

This section presents the various RPM programs identified within the participating health services. Pre-COVID programs included management for outpatients with chronic conditions, such as heart failure, diabetes, and chronic obstructive pulmonary disease (COPD), and acute Hospital in the Home (HITH) services. During the COVID-19 pandemic, RPM services expanded to include HITH for COVID-positive patients and monitoring using thermometers, pulse oximeters, and phone calls. As the pandemic persisted and health services adapted to the ongoing challenges, new and continued RPM programs included heart failure monitoring, programs for frequent emergency department attenders, and specific programs for post-operative monitoring, home-based sleep studies, and diabetes management. Additional RPM applications involved palliative care services for older Australians, aged care monitoring, a Cancer Service App for patients with prostate cancer, and outpatient RPM for wound management.

The barriers and facilitators to implementing Remote Patient Monitoring (RPM) in rural and regional settings are structured across micro, meso, and macro levels [29]. This multi-level approach highlights individual, organizational, and policy-related factors, offering a detailed perspective on the challenges and opportunities in these settings [29]. The identified barriers and facilitators are presented in Table 1, and key themes are summarized in the following section emphasizing how they operate at different levels to shape the implementation of RPM in rural and regional healthcare environments. Direct quotations from the semi-structured focus groups are included to provide depth and illustrate key findings.

**Table 1:** Barriers and facilitators in RPM implementation in rural and regional Victoria

Theme	Barriers	Facilitators
**Micro-Level (Health Professional and Patients)**		
**Patient Digital Literacy and Technology Readiness**	- Difficulty in engaging patients, especially older adults, due to low digital literacy and language barriers. - Patients are often not familiar with digital tools. - Language barriers further complicate training and engagement.	- Community-driven initiatives, such as telehealth parties, to bridge digital literacy gaps and familiarize patients with digital services. - Telehealth parties involve group sessions where individuals can learn and practice using digital health tools together, which can improve comfort levels with technology.
**Patients’ Personal Preferences**	- Patients’ preference for in-person visits and resistance to adopting digital tools for healthcare management. - Strong cultural preference for face-to-face interactions. - Distrust in digital solutions due to lack of personal touch.	- Combining digital and non-digital methods, such as telephone-based support, aligns with community preferences and serves as a bridge to digital engagement. - Telephone-based support provides an accessible starting point for patients, helping them transition to digital tools while respecting their comfort levels and preferences.
**Meso-Level (Health Care Organizations)**		
**Organizational Infrastructure**	- Lack of system interoperability, exacerbated by difficulties in transferring patients across departments or health services, impacts patient care. - Fragmented IT systems hinder seamless data sharing. - Inadequate digital infrastructure limits system efficiency.	- Investment in technological infrastructure, including the development of consolidated technology strategies and the implementation of effective ICT governance structures.
**Regional Workforce and Role Overload**	- Challenges in attracting staff with relevant expertise to rural and regional areas. - Difficulty in recruiting specialized digital health staff. - Existing staff being overburdened and spread thin across multiple roles.	- Many healthcare professionals in rural and regional areas demonstrate a strong commitment to engaging with technology to improve service quality and respond effectively to patients’ needs.
**Staff Training and Support**	- Insufficient training opportunities and lack of ongoing technical support decrease confidence in delivering RPM, exacerbated by difficulty in retaining specialized staff in rural and regional areas	- Comprehensive training programs and support systems for healthcare staff.
**Collaboration, Partnership, and Evaluation**	- Evaluations of implemented health interventions necessitate substantial resources and funding. - Resource constraints hinder continuous improvement efforts.	- Evidence-Based Integrated Approach: Collaboration with academic institutions for program evaluation and research. - Partnering with universities can provide necessary resources for evaluation.
**Macro-Level (Policy Makers and Health Systems)**		
**Government/ Policy**	Lack of a unified rural & regional digital health strategy - Digital connectivity issues in rural and regional areas may limit the reach and effectiveness of RPM. - Funding models do not adequately support rural digital health initiatives.	Development and implementation of equitable funding models supported by a unified digital health strategy to streamline digital health initiatives. - Subsidies for internet access and infrastructure improvements to enhance connectivity in rural areas.

#### Summary of key themes and illustrative semi-structured focus group excerpts

##### Theme 1: Patient digital literacy & technology readiness

Participants reported varied digital literacy levels, especially among older adults and highlighted other related factors that impact a patient’s ability to use RPM, including challenges like smartphone access and usage, language barriers, and issues with internet connectivity and device reliability in areas with poor digital infrastructure.

- *“We have to ensure that the person’s able to access that technology—do they have a smartphone or a device or a laptop? So, there’s a lot of assessment behind the scenes to make sure they can do it. The other issue is serviceability – so are they in a black spot area? Do they have internet? Do they have a landline even?”* (FG1)
- *“So I just think we need to be considering we’ve got 35 different cultural, linguistically diverse communities within region and how do we manage language differences?”* (FG2)

##### Theme 2: Patients’ personal preferences for digital and non-digital care

Healthcare staff perceived that some patients, particularly older adults and pregnant women, favoured face-to-face appointments for their personal connection over digital platforms. Clinicians, especially midwives, were cautious and preferred seeing their patients in person due to concerns about isolation and the specific needs of these demographics.

- *“We see a lot of isolation in our community. In my role, I work with many young women in midwifery, so we do a lot of phone calls, but we’re cautious. We like to see the woman in person. During COVID, women wanted to see their midwife in person. We definitely find benefit in seeing that woman in person.” (FG2)*

In rural areas, this preference is further emphasized by the geographical isolation and the lifestyle of patients, many of whom live on farms and experience significant loneliness.

- *“Some women are home on the farm, their husband is at work all day, and the kids are at school. The health appointment is sometimes their only outing, and it’s an enjoyable experience for them.” (FG2)*

However, healthcare staff perceived that other populations, such as those in early parenting, showed a notable openness to digital engagements.

- *“For early parenting, the demographic is very different. They appreciate the convenience of filling out information online before coming in, if it will reduce their consult time. This approach is more appealing to new moms compared to the elderly.” (FG5)*

##### Theme 3: Lack of integration and interoperability

Participants emphasized the critical need to integrate RPM technologies into existing healthcare systems and clinical health records. Addressing interoperability issues was considered essential to enhance RPM efficacy, alongside calls for upgraded technological infrastructure and governance. Challenges in accessing and sharing data between regional healthcare settings were also discussed by participants.

- *“If that person then was being monitored at home and then presented to an ED [Emergency Department] close by, that information is sitting in a record that the other health service might not be able to access. So, there is that integration, that interoperability between whatever programs we use and wherever we keep our data for our patients.” (FG3)*

Participants noted that poor interoperability and system integration sometimes led health services to reject RPM technologies entirely.

- *“‘No, we will not be using [the RPM technology].’ Healthcare providers had no alternative [technology], but they were more satisfied with having a phone call and having the person self-monitoring at home and talking to that person and then putting that information in their own medical record. [Healthcare providers] felt better about that.” (FG3)*

The issue was further complicated by the management of digital interventions, which sometimes involved multiple stakeholders with differing approaches.

- *“We’ve got three people [stakeholders] looking at something [digital interventions] that requires a system but nobody’s talking, so we might end up with three systems when one system could actually do the job for everybody.” (FG1)*

##### Theme 4: Funding and resource allocation

Participants emphasized the need for comprehensive and integrated funding strategies to support the implementation and sustainability of RPM programs, advocating for alternative funding approaches that avoid the limitations of fragmented and isolated funding sources.

- *“We don’t have resources for quality improvement. When discussing [lobbying for] funding, we need resources to [evaluate] remote monitoring as a program or model of care, [including] tools that support it and an understanding of [available] digital technologies and their capabilities.” (FG4)*

The challenge of resource allocation was compounded by issues in governance and fragmented funding structures.

- *“I think since we haven’t had proper governance [in place], the other problem is that we get funding from the department [of health]. Then we’ve got siloed funding [allocated for specific purposes only], which contributes to the whole problem of progressing digital health in a strategic sort of way.” (FG5)*

##### Theme 5: Collaboration, and evaluation

Participants emphasized the value of collaboration between health services, primary care, academic institutions, and other stakeholders in developing evaluating, and improving RPM services. While existing partnerships, such as those with universities, have been beneficial, participants highlighted the need to strengthen evaluations, by incorporating evidence-based strategies and increasing consumer engagement.

- *“Yeah, but we probably don’t pay enough attention to evidence-based literature as to why [current practices] is the way they are. So, I think that would, if anything, keep us accountable to make sure that we are paying attention to the evidence base. I mean we looked at the data, mostly around funding and cost in the hospital admission, but we haven’t looked in a lot of detail around consumer feedback and consumer engagement [for RPM programs]—that’s an opportunity for consumers to actually really contribute to the development of [these] intervention[s].” (FG4)*

Gaps in feedback and engagement for rural and regional consumers were also highlighted, including the lack of evidence-based strategies tailored to the unique needs of the region.

- *“[We aren’t] thinking more strategically [about RPM] if it is a model that will help us into the future. Almost like a proof of concept, we conduct the initial project or pilot and then make decisions [about broader implementation] after that. Also, considering what the financial cost and the benefits are, we do have the cost-benefit analysis of [the RPM implementation]. Once again, not all our projects have that [for informed decision making].” (FG1)*

##### Theme 6: Staff training and support

Comprehensive training and support in digital health technologies and RPM tools for healthcare staff were emphasized as crucial for boosting their confidence and capabilities in delivering RPM services effectively.

- *“Like, we’re all RN’s [Registered Nurses] and we technically have done the same university degree, but obviously some got a lot more training than other colleagues [in Digital Health]. We already see that within our own team and our own level…Now, if we’re looking to save cost [on monitoring tasks]—since it’s just monitoring numbers, can an EN [Enrolled Nurse] do it [monitor RPM data]?… So, yes, there is a recognition that this is a specialty area [Digital Health/RPM] that requires people with a minimal amount of qualification to be able to monitor.” (FG3)*

Participants noted disparities in training opportunities and resources, especially highlighting how RPM roles and objectives have become blurred since COVID, impacting rural settings more significantly.

- *“I think it’s become really blurry [RPM program roles and objectives have become] since COVID, because it was all really geared towards COVID. Maybe redefining exactly who they are [RPM programs] and what they are [digital tools] and what they can do probably hasn’t been revisited with external people [external stakeholders] that may refer outside of our immediate space.” (FG4)*

## Discussion

The results of this study highlight that digital transformation in healthcare is a pervasive technological shift presenting substantial challenges for rural and regional communities [30]. Healthcare professionals reported micro, meso, and macro-level challenges intricately linked to digital determinants of health—factors such as digital literacy, internet connectivity, device affordability, and the availability of digital health tools and resources [31, 32]. These factors highlight potential disparities in digital health access and outcomes between rural and metropolitan areas, particularly for patients with low digital literacy and language barriers. The lack of resources to support patients with low digital literacy and language barriers underscores the need for tailored approaches to improve RPM implementation. Healthcare staff also discussed different levels of skills and training in RPM, with participants stressing the need for specific training and the recognition of the special skills required for RPM tasks, particularly in rural settings where it may be difficult to attract and retain staff [1]. These broad challenges associated with digital health in rural areas, consistent with previous studies, have been well-illuminated in the literature [1, 2, 16, 19–21, 33].

The healthcare professionals in this study provided several strategies that may mitigate the unique barriers facing RPM implementation in rural and regional Australia, that fell under three themes: (1) the necessity for effective strategies to ensure everyone can access and benefit from RPM regardless of their location, (2) the critical need to bridge the infrastructure and technological divide by investing in IT infrastructure that may elevate rural areas to the same standards as metropolitan regions, and (3) the urgent need for sustainable and targeted funding models to ensure the equal distribution of resources and support the long-term viability of RPM initiatives. These results suggest that there may not be a single, universally applicable strategy to address these challenges; instead, the implementation of remote patient monitoring (RPM) in rural and regional settings may require a multifaceted set of strategic actions [34]. The three themes are discussed in more detail below.

### Accessible RPM

#### Acceptability

Contrary to findings from the COVID-19 era, [16, 18–21], our qualitative research, encompassing a broad spectrum of RPM services, suggest that RPM may not be universally suitable across all clinical scenarios. Importantly, the findings of this study bring to focus a nuanced perspective among healthcare providers in rural and regional settings: while they use and acknowledge the potential benefits of RPM, they may prefer face-to-face interactions with specific patient groups and under certain medical conditions. For instance, participants mentioned a reluctance toward digital modalities in areas like Midwifery (theme 2), where clinicians preferred more traditional, in-person consultations, reflecting the value placed on personal interaction in rural healthcare contexts. Conversely, in the post-partum period, there was a marked preference among new mothers for RPM, welcoming the use of tools such as questionnaires for data collection if these could replace or shorten the duration of clinic visits. Consistent with findings from other research [20], clinicians perceived targeted data collection for RPM as practical and effective for certain conditions like congestive heart failure, diabetes, asthma, weight management, and post-surgery recovery. This variability suggests a need for RPM programs to be flexible and tailored, considering factors such as the complexity and sensitivity of the health condition and the need for physical examinations.

An important consideration is the distinction between initial digital interactions and established patient relationships [15]. In rural settings, where personal interaction is reportedly highly valued, initial face-to-face consultations may be crucial for building the necessary trust and rapport [1]. This foundation can make subsequent digital interactions more acceptable and effective. Therefore, hybrid models, which combine initial in-person visits with digital follow-ups, could offer a more effective approach for RPM programs in rural areas. This distinct shift has been emphasized following the onset of the COVID-19 outbreak [15]. Nevertheless, emerging research has raised concerns about the potential mental health risks associated with digital services; suggesting that these services might discourage physical activity since patients are not incentivised to leave their homes for appointments, which could negatively impact their overall well-being [22]. Notwithstanding, these dynamics may warrant further investigations to ensure the efficacy of RPM programs. Additionally, implementing an RPM assessment or checklist to identify suitable patients could enhance the effectiveness and efficiency of these programs. By systematically evaluating patient suitability, healthcare providers may ensure that RPM is appropriately matched to individual needs and conditions, improving overall outcomes [35].

#### Affordability

Ensuring equitable access to RPM technologies requires addressing key barriers such as affordability, particularly for vulnerable populations in rural areas [36, 37]. Participants also pointed out that certain patient groups, like older adults, might hesitate to use RPM due to the costs associated with data plans or internet services. This aligns with findings from a telehealth study, which emphasized how costs significantly influence consumer decisions [11]. Participants expressed a preference for cost-effective RPM solutions, as higher out-of-pocket expenses remain a significant barrier in rural areas. This indicates that RPM initiatives that reduce or eliminate patient costs are likely to see higher acceptance and use among the intended populations. Importantly, existing research has shown that older people are not necessarily less likely to use RPM. Studies indicate that with adequate support and resources, older adults can effectively engage with RPM technologies [38]. Findings highlight the importance of integrating telemonitoring into care models to support patients with complex conditions, regardless of age [38]. This suggests that barriers to RPM use among older adults can be mitigated through supportive measures, such as providing tools and training for self-management [38]. Nevertheless, it is essential to further investigate these concerns, indicating a need for targeted discussions with consumers such as older people living in rural and regional areas to better understand their specific barriers and preferences, particularly related to affordability [15, 38]. Additionally, it could be worth considering the broader needs of end-users, including both patients and healthcare providers, when designing inclusive and effective RPM programs, ensuring the technology is also evidence-based [15].

### IT infrastructure

The findings of this study suggest that rural and regional areas could significantly benefit from enhanced internal information technology (IT) support that matches the comprehensive systems found in metropolitan hospitals [37]. Strengthening these capabilities is vital to avoid fragmented services, delays in addressing urgent IT issues, and disruptions in RPM system operations. Achieving this enhancement, however, is complicated by varying levels of dedicated IT support in rural hospitals. In certain cases, coordination with larger referral centres becomes difficult, resulting in data silos and communication breakdowns [39]. This means that essential patient information is often trapped within isolated systems and not readily accessible to other hospitals that need it, leading to inefficiencies and potential errors in patient care. Additionally, these isolated systems raise significant concerns about data privacy and security [39]. When hospitals lack dedicated internal IT teams, they may rely on external or outsourced IT services, which may not prioritize patient confidentiality to the same extent as internal teams. This reliance increases the risk of data breaches and unauthorized access, making it crucial to strengthen internal IT capabilities to safeguard patient data effectively [39]. These organizational issues are especially evident in rural and regional settings, where limited local resources and the logistical challenges of maintaining advanced IT infrastructure in remote locations exacerbate the problem [40].

Moreover, systemic barriers such as inadequate infrastructure and fragmented governance further complicate efforts to implement reliable IT systems. Evidence from other sectors, including agriculture, banking, and education, highlights similar challenges, where systemic inefficiencies and delays hinder operational delivery and service effectiveness [37]. For example, insufficient IT support in rural banking branches can lead to service delays and increased vulnerability to security breaches [41]. In education, the digital divide between urban and rural schools exacerbates educational inequalities, affecting students’ access to quality learning resources and support [41]. These cross-sectoral challenges suggest the need for a cohesive, cross-sectoral strategy to address infrastructural deficiencies in rural and regional areas [41, 42]. Such a strategy could enhance healthcare delivery through improved RPM systems and bolster overall community development by integrating solutions across various sectors.

Evidence on health investments in Australia highlight significant disparities in per-capita health spending between urban and non-urban citizens, with a shortfall of $848.02 per person in 2020–21, amounting to a total shortfall of $6.55 billion [42]. The inequitable investment in IT infrastructure means these areas often lack the necessary foundation to support innovative digital solutions. Participants discussed challenges related to fragmented IT systems and limited alignment with local needs. Moreover, many RPM studies reported in the literature are conducted within metropolitan healthcare settings [16, 17] and globally [18–21]. While these systems may reflect the priorities and resources characteristic of urban healthcare environments, their applicability to rural and regional contexts remains uncertain [43]. It stands to reason that interventions studied or tested within metropolitan settings may not fully account for the unique challenges faced by non-urban populations [15]. Designing these interventions with rural and regional contexts in mind from the outset, rather than simply adapting metropolitan solutions, may ensure they are better suited to local needs [15, 44]. These discrepancies suggest that tailored solutions and equitable investment could play a crucial role in improving healthcare outcomes in rural settings.

### Healthcare funding

A significant barrier identified was the misalignment of funding models between metropolitan and regional areas. Participants noted that the funding periods are typically too short to support the long-term investments in workforce and facilities necessary for creating sustainable RPM programs. This lack of sustained investment could also undermine efforts to provide the certainty required to attract and retain clinicians in non-urban areas [45]. Moreover, current Australian funding models for metropolitan hospital is through activity-based funding and fee-for-service funding, with block funding common in rural and regional setting, making it challenging to get a clear picture of the disparity in health expenditure [42]. Reports also show rural Australians have a poorer health status, and even before accounting for the increased cost of health service, receive significantly less funding per capita than their urban counterparts [42]. In theory, RPM programs funded through block grants can achieve budget neutrality or even cost-savings by preventing emergent hospitalizations from exacerbation of chronic disease, which often involve lengthy stays whose costs exceed the standard reimbursement [22, 46]. However, in practice, small hospitals may often hesitate to invest in RPM for chronic disease management, especially during periods of budget cuts, due to the uncertainty of outcomes for small programs. Furthermore, RPM programs that are cost-effective in terms of patient outcomes and quality of life might not meet the threshold for budget neutrality or cost savings [22].

Nevertheless, in metropolitan areas, which operate on activity-based funding, RPM programs are often not classified as National Weighted Activity Units (NWAUs)—a measure used to allocate funding based on the complexity and type of hospital activity—potentially preventing direct reimbursement for remote monitoring activities [22, 35]. This classification issue in metropolitan areas highlights the broader systemic challenges faced by both urban and rural health services under the current funding models [22]. Although federal and state governments appear to recognize these challenges and have invested in programs to address the health issues in rural Australia, these efforts may not have fully overcome the disparity in health outcomes [42]. The current pattern of health service use suggests a missed opportunity for early intervention and preventative healthcare [42]. Additionally, current funding models and service delivery arrangements create significant barriers to workforce recruitment and retention, further exacerbating the funding shortfall [47].

To effectively address this inequity in healthcare and health outcomes, the specific barriers to delivery and the shortcomings of the current approach need to be acknowledged [1]. A one-size-fits-all approach to funding arrangements may not be effective in addressing the complex challenges faced by non-urban communities [12, 42]. Tailored funding models that address the specific needs and challenges of rural Australia may prove promising [22, 42]. Additionally, alternative mechanisms for RPM reimbursement, such as bundled payments (payment for well-defined care pathways spanning multiple care settings over long periods) and capitation payments (care for a patient over a defined period of time where the provider is responsible for all health services consumed). Participants in the study unanimously emphasized the necessity for further evaluations of RPM programs. Economic modelling and additional assessments were recommended to determine the sustainability and effectiveness of RPM across various patient populations, and to identify payment models that could encourage high-value care pathways. However, implementing such models may face challenges, including the need for coordinated policy changes and the risk of uneven resource allocation, particularly in rural settings where resources are already limited [35, 42].

## Implications

### Research

This research contributes to the existing literature in the following five ways. *First*, it emphasizes the critical need for equitable digital health interventions by examining the barriers and enablers to RPM uptake in rural and regional areas. This focus on underserved populations aligns with national priorities to address rural health inequity [42]. *Second*, this study offers timely insights into the use of RPM since the onset of the COVID-19 pandemic, reflecting the rapidly evolving landscape of digital health technologies with recent findings. *Third*, it broadens the scope of understanding by simultaneously examining RPM use across various health conditions, extending beyond the singular focus on conditions like COVID-19. This comprehensive approach offers valuable insights into a wider range of chronic diseases and health management scenarios. *Fourth*, employing the Consolidated Framework for Implementation Research (CFIR) as a conceptual lens allows for a systematic identification and addressing of the complex barriers and facilitators influencing RPM adoption in rural and regional healthcare settings. The barriers and facilitators are structured across micro, meso, and macro levels, highlighting individual, organizational, and policy-related factors. This approach of distillation offers a detailed perspective on the challenges and opportunities across multiple layers, thereby enhancing the robustness and relevance of the study’s outcomes. *Fifth*, the study generates actionable recommendations aimed at improving access to RPM and promoting digital health equity and for vulnerable populations, such as older adults, in rural and regional areas. These recommendations are informed by the perspectives of healthcare professionals, which are crucial for effective policy and practice interventions.

### Practice

In general, the results of this research suggest that fostering RPM in rural and regional health regions can be achieved by improving access. Additionally, consideration should be given to end-user preferences, such as the flexibility of in-person versus online consultations, and the complexity and sensitivity of the health condition. The success of RPM in non-metro regions also hinges on having advanced IT infrastructure and sustainable funding to support these services. Implementing these strategies requires bold and coordinated actions that systematically involve all stakeholders in the rural and regional health ecosystem.

Digital interventions, such as RPM, operate within a complex ecosystem influenced by micro, meso, and macro-level factors [29]. Therefore, digital health initiatives must address these multi-level influences to be effective. Key stakeholders are advised to reconfigure their interventions and deployment strategies by adopting a new mentality oriented towards the unique challenges of rural and regional areas. As discussed above, ‘one size does not fit all,’ particularly in rural and regional contexts [12]. If key stakeholders continue to treat non-metro regions as secondary or apply solutions that were designed and tested in metro regions without adaptation, the risk of limiting implementation of new digital models of care becomes high. This indicates that interventions crafted for metropolitan settings may not seamlessly address the unique challenges of rural and regional areas.

In light of these findings, this research makes the following invitations: [15, 44]. *Meso-Level Stakeholders*—This research calls on meso-level stakeholders, including healthcare organizations, to adopt tailored RPM implementation approaches to address sociotechnical barriers faced by health professionals and patients [15]. Partnerships with research organizations, such as universities, can leverage rural and regional knowledge domains, helping to develop specific actions and recommendations that improve work quality, health outcomes, and RPM utilization. *Micro-Level Stakeholders*—Regional dialogue among healthcare organizations should create benchmarks for micro-level experiences and facilitate information exchange across health services. A co-designed platform may subsequently be made available to all.

### Policy

Finally, this study invites macro-level stakeholders, including policy makers in the rural and regional innovation system to commence discussions to develop equitable intervention, along with region-specific RPM implementation frameworks, centred on the following three pillars: (1) *Technological and Digital Infrastructure development*- Implement policies to develop a comprehensive technological and digital infrastructure system, ensuring widespread access to RPM. This includes mandating affordable Internet access in rural and regional areas. (2) *Increased Funding*- Policy makers should formulate funding policies that allocate equitable financial resources specifically for RPM implementation and digital health transformation. This could involve creating dedicated grants or subsidies for rural healthcare providers and incentivizing private investments in these areas. Importantly, these initiatives should ensure parity with metropolitan regions [33]. Lastly, (3) *Stakeholder Collaboration*- Develop and enact policies that promote collaboration between various stakeholders to strengthen regional ties. This study points out creating frameworks for stakeholder engagement, establishing regional health committees, and fostering lifelong learning pathways to ensure continuous professional development and quality improvement. Moreover, fostering ‘table talks’ as strategic and policy initiatives will support collaboration and communication among all rural and regional actors, including policymakers, healthcare providers, technology experts, academics, community representatives, and patients in RPM design and implementation.

## Limitations

While this study offers valuable insights, it is important to note its limitations. The perspectives gathered were from healthcare staff and not directly from consumers, potentially overlooking varied patient experiences with RPM. Additionally, findings are drawn from five health services in Western Victoria, Australia, reflecting the experiences of participants within these settings. Participants were selected based on their involvement with RPM programs, the findings may not fully reflect the perspectives of healthcare staff with little or no experience with RPM. Although, ‘member checking’ was employed to enhance credibility by verifying findings, this process could still harbor biases due to the subjective interpretations of participants. Participants’ experiences included those from the COVID-19 pandemic, which could influence the findings, as responses may reflect unique pandemic-related constraints such as lockdowns, possibly skewing perceptions of RPM’s effectiveness. Bias may also be present in the method of convenience sampling, which may not be representative of the wider rural and regional population. The study focused on capturing professional diversity rather than collecting individual demographic details such as age or gender, which may limit the granularity of participant insights. However, the study included participants from five hospitals across a wide geographic area, ensuring that a diverse range of organisational perspectives were represented. While these findings provide valuable insights into similar contexts, their applicability to other areas or countries at different stages of digital health transformation should be carefully considered in light of contextual differences.

## Conclusions

The complex and distinctive challenges of rural and regional health service ecosystems recommend a tailored approach to implementing RPM. The application of findings from RPM studies predominantly conducted in urban settings [16, 18–21, 48], to rural environments is fraught, primarily due to disparities in technological infrastructure, digital literacy, and healthcare resource distribution. Our findings indicate that a context-sensitive implementation strategy for RPM is essential. This strategy must be accompanied by focused policy initiatives and adaptable resource allocation to achieve equitable health outcomes across varied geographic landscapes. Addressing these unique challenges through targeted policy reforms and resource distribution could play a significant role in supporting the successful implementation and sustainability of RPM in rural and regional areas. Future RPM programs, services, and technologies could benefit from co-design with rural and regional stakeholders, including consumers, to ensure they meet unique local needs. In addition, future research could investigate the experiences, preferences, and barriers faced by patients using RPM services in these contexts. Comparative studies are needed to explore disparities in RPM uptake, accessibility, and outcomes between metropolitan and non-metropolitan areas. Longitudinal studies may evaluate the impact of RPM on health outcomes and healthcare utilization over time, while targeted research could assess the financial viability, long-term sustainability, and effectiveness of specific policy interventions in resource-constrained settings. By embracing these recommendations, we may take steps towards a more equitable and effective healthcare system that better addresses the needs of Australians in diverse geographic locations.

## Supplementary Information

Supplementary Material 1.

## Data Availability

The datasets used and/or analyzed during the current study are available from the corresponding author upon reasonable request.
