# Supplementary material for: Challenges for remote patient monitoring programs in rural and regional areas: a qualitative study
Source: BMC Health Serv Res. 2025 Mar 13;25:374. doi: 10.1186/s12913-025-12427-z (PMC11905676; doi:10.1186/s12913-025-12427-z)
Supplement: Supplementary file 1 — Supplementary Material 1. [file 12913_2025_12427_MOESM1_ESM.pdf]

Interview Schedule: DELIVER

DELIVER
Research for better healthcare closer to home

Overview of Focus Group and Interview Schedule: Remote Patient Monitoring (RPM) implementation

The purpose of the focus groups and individual interviews is to reflect on participants' experiences with remote patient monitoring (RPM) in their respective organizations. All comments will be anonymized, and participants' identities will not be disclosed in any publications. Sessions will be recorded for transcription and analysis, with all data managed according to institutional research policies.

Focus Group Guidelines:

- Participants are encouraged to share their experiences, and differing viewpoints are welcome.
- If participants speak at the same time, responses may not be fully captured.
- Each session, whether a focus group or individual interview, may last up to 90 minutes, with a scheduled break halfway through.
- Any additional information participants wish to provide after the focus group is also welcome.

Introductions

- Participants will introduce themselves and indicate whether they hold a clinical or non-clinical role.

Interview Questions

1. Please describe the type of remote patient monitoring services and patient groups, if any, your organisation supported ***before COVID-19***.
2. Please describe the type of remote patient monitoring services and patient groups, if any, your organisation supported ***during or after COVID-19.***
3. If you have an example, please describe the type of remote patient monitoring services and patient groups your organisation has specifically ***designed for older Australians.***
4. Are you delivering virtual palliative care services and does that contain any RPM?
5. What are the ***barriers*** to RPM in your organisation? (If RPM is not supported, we need to focus more on these barriers: Core needs and internal motivations, technological readiness, staff and patients' acceptance and use, IT skills/training/learning capability of the organisation, organisational engagement with existing technology and overall organisational readiness)
6. What are ***facilitators*** or supporters of RPM in your organisation?
7. Now thinking about the technology, can you share with me some information about the ***modes of RPM*** used in your organisation. I want to ask about three things: do you have virtual visits; are you using patient surveys or PROMS; and are you able to perform vital sign monitoring through biometric devices? (Sinn et al, 2022) Would you like to?
8. Now thinking about ***RPM management*** at a clinical level, can you let me know how RPM is staffed and who assumes responsibility for the service?
9. Thinking about your most mature example ***how often*** are RPM services used or do you have a 24/7 service, and what happens when something needs to be ***escalated***?

10. What are the biggest challenges your patients face in accessing / using your RPM service? Are black spots without internet connection an issue? Do patients mostly use their own devices or ones you provide and is there education needed to upskill patients on how to access RPM?
11. What is your implementation process for a newly introduced technology (Probe: people, specific guidelines, internal policies, or strategies)?
12. Is there a preference in your organisation for out-of-the-box or turnkey RPM or do you build and maintain RPM yourselves?

BREAK?

13. Who ***onboards patients*** into the RPM programs, and do you have ***dedicated management*** or administration staff to manage the RPM services?
14. Thinking about your most mature RPM program, how ***interoperable*** is the RPM in your organisation?
15. Thinking about your most mature RPM program, how is the ***data processed*** and where is it ***stored***?
16. On the theme of data, can you please describe the level of continuity of the ***data flow***, for example do you use store and forward, or is the data continuously synced?
17. Thinking about your most mature RPM programme, if you are involved in using/running the service, how often do you have to use ***workarounds*** to get your job done?
18. How is RPM ***monitored and evaluated*** at your organisation?
19. Does ***hospital leadership*** to evaluate these programs, if yes why?
20. Now thinking of the current state of your organisation, what RPM solutions would you like to ***enhance***?
21. Now thinking of your organisation in 5 years' time what RPM programs has your organisation invested in? ***What is the priority for your organisation?***
    a. Probe: What specific benefits – clinical, service or otherwise – would this drive?

ENDING: Thank you for your time and insights. Are there any final comments before we conclude?

Note: CFIR Framework and Interview Guide
The interview questions were designed to align with the Consolidated Framework for Implementation Research (CFIR), addressing its key domains: **Intervention Characteristics** (e.g., questions 1–4, 7, 15, 16), **Outer Setting** (e.g., questions 10, 12, 20), **Inner Setting** (e.g., questions 5, 6, 8, 9, 13, 14), **Characteristics of Individuals** (e.g., question 10), and **Process** (e.g., questions 11, 17, 18, 19, 21).
